# The finite element neural network method to simulate two dimensional partial differential equations and perform parameter identification

**DOI:** 10.1038/s41598-026-46707-3

**Published:** 2026-04-18

**Authors:** Mohammed Abda, Lucas Berthet, Mohsen Hamedi, Dani Hibatullah, Elsa Piollet, Christopher Blake, Bruno Blais, Frédérick P. Gosselin

**Affiliations:** 1 Laboratory for Multi-scale Mechanics (LM2) of the Department of Mechanical Engineering, Polytechnique Montréal, Montréal, Canada; 2 Maya HTT, Montréal, Canada; 3 CHAOS Laboratory of the Department of Chemical Engineering, Polytechnique Montréal, Montréal, Canada

**Keywords:** Engineering, Mathematics and computing, Physics

## Abstract

Neural networks (NNs) have received growing interest in engineering due to their ability to assimilate high-dimensional data and provide accurate approximations for complex systems. Nonetheless, classical numerical methods remain the benchmark for reliability and accuracy, backed by rigorous development over decades. Merging the strengths of the finite element formulation with physics-informed NNs (PINNs), the finite element neural network method (FENNM) opens new venues for approximating partial differential equations (PDEs). FENNM is based on the Petrov–Galerkin framework, where the NN provides the global nonlinear space of solutions, whereas the test functions are the nonvanishing Lagrange shape functions. Compared to VPINN, *hp-*VPINN, *cv-*PINN, and FastVPINN, FENNM’s weak-form explicitly includes flux terms at the elements’ interfaces and naturally incorporates Neumann boundary conditions within the residual loss function, improving the training stability and adaptability to real-world applications. We extend FENNM to two-dimensional domains, with the second dimension representing space, time, or a parameter. The method naturally integrates time and parameter spaces for design optimization, offering advantages over the deep energy method and discrete finite element method inspired NNs. We further showcase FENNM’s capability in local mesh refinement, vector-valued PDEs, inverse problems, and complex geometries with irregular elements.

## Introduction

An accurate and efficient approximation of PDEs is essential for engineering applications and applied science. This becomes more demanding in applications that require real-time simulations^1^, development of digital twin technologies^2^, and when dealing with ill-posed systems such as incomplete boundary conditions. Traditional FEMs are the standard of reliability for a wide range of practical applications^3,4^. However, FEM becomes computationally challenging for large-scale applications, optimization problems, and parametric identification problems. Over the past decade, PINNs have emerged as a promising paradigm for solving PDEs^5,6^. PINNs seamlessly integrate data into the solution process, treating compliance with the underlying PDEs as a soft constraint within a combined loss function. This unique approach makes PINNs particularly well-suited for tackling inverse problems. However, they face their own challenges, enforcing boundary conditions usually gives rise to competing loss terms, hindering their convergence. Integrating the strict foundation of FEM with the flexibility and data-driven adaptability of PINNs in a hybrid solver paves the way for addressing these limitations while harnessing the advantages of both methods.

FEM has undergone meticulous refinements over the decades, making it the standard approach for many engineering applications. The robustness, computational efficiency, and versatility of FEM makes the method appropriate for addressing problems of complex industrial geometries and boundary conditions^3,4^. This is because the FEM approach allows the reformulation of PDEs as a system of algebraic equations. However, it requires prior knowledge of the parameters, loadings, and boundary conditions, and requires a well-posed problem^7^. Nevertheless, one of the current challenges in FEM is that confrontation with experimental measurements can only be performed once the simulation is complete^8^. Experimental data cannot be incorporated into the simulation.

The PDE solution can be formulated as an optimization problem in which a NN iteratively approximates the solution using a predefined loss function^9,10^. As universal nonlinear function approximators^9,11–14^, NNs have been explored as alternative approaches to approximate PDE solutions for inverse problems, and for parametric identification problems^8,15^. PINNs use strong-form PDE residuals to construct the loss function, which, for given boundary and initial conditions, restricts the output to the solution without requiring high-fidelity data^10,14,16,17^. Once trained, PINNs generate the solution as a function defined in the domain without requiring a computational mesh^18,19^. Moreover, they can effectively address inverse problems by seamlessly integrating sparse or noisy data into the training process^20^. However, they are sensitive to initialization and require complex hyperparameter tuning to balance competing loss terms^20^. In addition, they have convergence and accuracy problems with stiff PDEs and solutions with sharp transitions in space^21,22^.

The performance of PINNs can be improved by using the variational form of the PDE as a residual loss function. This is because the variational formulation lowers the order of the PDE while easing regularity requirements, making it more effective at handling stiff problems, steep gradients, and singularities. This, in turn, reduces computational expenses by minimizing the number of backpropagation processes needed, as noted in ref^10,14^. In the variational PINN (VPINN)^10^, the weighted residual of the PDE is used to construct the loss function. The weighted residual here is based on the Petrov–Galerkin framework, where the NN represents the nonlinear space of solutions, and the test functions are a combination of Legendre polynomials with vanishing values at the boundaries. The *hp-*VPINN method extends VPINN to include non-overlapping elements, each associated with test functions of varying orders^14^. Two alternative TensorFlow-based methods have been introduced to improve the computational efficiency of *hp-*VPINN. The convolutional VPINN (*cv-*PINN)^23^ uses convolution operations to evaluate the strong-form weighted residual of the PDE instead of for-loops, and FastVPINN^24,25^ uses Matrix-Vector product reformulation to evaluate the weak-form weighted residual of the PDE and handles complex geometries. However, the test functions in VPINN and its variants disappear at the boundaries of the elements, which simplifies implementation, but also causes a loss of flux information^26^. Therefore, with vanishing test functions, VPINN and its variants cannot impose natural boundary conditions and intermediate loads within the residual loss function as demonstrated by Abda et al.^26^. Furthermore, since the NN is forced to compensate implicitly the flux terms in these methods, they require an excessive number of test functions and quadrature points, imposing an avoidable computational overhead as implemented in^10,14,24,25^.

The variational formulation can also be obtained by minimizing the PDE energy functional as a loss function. Inspired by the Ritz method^4^, the Deep Ritz method^27^ is the first to use the variational form of the PDE as a loss function, where the NN approximates displacements at randomly sampled collocation points within the domain using stochastic gradient descent (SGD). This work is further extended to use the total mechanical energy of linear systems using the deep energy method (DEM)^28^. Based on the principle of minimizing potential energy, the DEM is further modified to include nonlinear PDEs^29^ and to solve for problems of local stress concentrations^30^. Evaluating the loss function on the elements’ nodal points in an FEM discretized domain improves the NN predictions as demonstrated in DeepFEM^31^ and the neural network-augmented differentiable FEM (NNDFEM)^32^. The authors use FEM shape functions to approximate the displacement of any point inside the elements and define the energy component derivatives of the displacements as a function of the shape functions which improves the prediction accuracy. However, DEM has several limitations that hinder its applicability and generalizability. First, the energy functional is not available for all types of PDEs. Second, Dirichlet boundary conditions are challenging to impose and require problem-specific treatments. Third, the NN does not have direct knowledge of the PDE, necessitating traditional post-processing to derive quantities like residuals and discretized higher-order operators, which are necessary for local mesh refinement techniques, which are typically less precise than the results produced through automatic differentiation (AD)^33^. Fourth, employing shape functions to compute displacements confines the solution space that the NN can explore.

The discretized FEM formulation can be used to construct the variational form of the loss function. In the FEM-enhanced neural network (FEM-NN)^8^, the loss function is the algebraic system of discretized Galerkin FEM equations and the NN is trained to solve for nodal variables. The advantage of this method is that it requires one loss function, as the boundary conditions are included in the stiffness matrix and the forcing vector. For ill-posed problems, the mesh-based PINN (M-PINN)^20^ maps discrete observed data within the domain to the FEM mesh nodes, then it is trained to match the mapped nodal variables^20^. Subsequently, the finite element-integrated NN (FEINN) is developed using the Galerkin framework to address the elastic and path-dependent elastoplastic problems in solid mechanics^34^. Using a convolution NN (CNN) in finite-element-based PINN (FEPINN), the method is extended to include variable and arbitrary domains^15^. This is done by mapping the nodal points from the physical domain to the local domain using stencil convolution operations. According to ref^15^, the mapping loses its accuracy with high-order shape functions. However, using the discretized FEM formulation comes with several challenges. First, the solution is approximated at nodal points, which demands using either denser meshes or higher-order shape functions to achieve mesh-independent approximations, which increases the loss function complexity. Second, the Galerkin formulation restricts the nature of the solution space to a linear combination of the shape functions and nodal values. Third, the NN does not have direct knowledge of the PDE, which requires further post-processing as explained in the DEM case. Fourth, up to this point, this approach has primarily been applied to static problems, and its extension to time-dependent and parameter optimization scenarios remains an open question.

As highlighted above, the solution of the weak form, whether derived through the variational approach with vanishing test functions at the element boundaries, the energy functional, or the discretized FEM formulation, using PINNs presents notable limitations that restrict their full potential. In this regard, Abda et al.^26^ proposed to solve a weak-form FEM formulation using PINN called the *finite element neural network method* (FENNM) in one-dimensional domains. FENNM is based on the Petrov–Galerkin framework, where the nonlinear NN output represents the global trial solution that is used to compute the PDE residuals, while the nonvanishing FEM shape functions are the test functions. Using the nonvanishing FEM shape functions ensures that the boundary terms in weak-form of the PDE do not vanish and are explicitly learned. In contrast to earlier methods discussed^10,14,23–25^, these boundary terms act as constraining terms that help the NN guide itself towards the solution, thus improving the optimization. Moreover, the boundary terms can be used for imposing Dirichlet and Neumann boundary conditions within the residual loss function using a single NN, thus minimizing the number of competing loss terms and the complexity of the NN model. The weak-form of the residual loss function is then integrated using the Gauss quadrature rule. Finally, FENNM uses TensorFlow convolution operations^35^ to perform summations over Gauss points for all elements in the domain, which are done in parallel for all test functions. Results reported by Abda et al.^26^ show that the explicit imposition of flux terms leads to a complete residual loss function, which can be generalized to all classes of test functions. In addition, the number of test functions and quadrature points required is significantly lower when approximating the same solution using non-vanishing test functions^26^.

Here we propose to extend the FENNM framework^26^ to two-dimensional problems. We show that FENNM can solve problems in space, time, and parameter space. Combining the strengths of both FEM and NNs allows solving problems with complex geometries, as well as forward and inverse problems. The focus of this work is to bridge the conceptual gap between PINN and FEM methodologies rather than compete with FEM in terms of computational speed.

The remainder of the paper is organized as follows. Section "Methodology" presents the development of FENNM and a detailed explanation of how the method works. Section "Case studies" shows through five numerical experiments how the FEM shape functions can be used in the FENNM formulation to solve time-dependent PDEs, local mesh refinement, parameter-space for optimization problems, vector-valued PDEs in fluid mechanics, inverse and parametric identification for heat transfer problems, and complex geometries with irregular meshes. Final remarks, conclusions, and future work are presented in Sect. "Conclusion".

## Methodology

### Construction of the FENNM loss function

Consider the following problem

1a $$\begin{aligned} L^{\textbf{q}}[u(\textbf{x},t)]&= f (\textbf{x},t), \quad (\textbf{x},t) \in \Omega \times (0,T], \end{aligned}$$

1b $$\begin{aligned} u(\textbf{x},0)&= g(\textbf{x}),\quad \textbf{x} \in \Omega \end{aligned}$$

1c $$\begin{aligned} u(\textbf{x},t)&= h(\textbf{x},t),\quad (\textbf{x},t) \in \partial \Omega \times (0,T], \end{aligned}$$

where the operator $L^{\textbf{q}}$ represents a differential operator depending on some parameters $\textbf{q}$, and $u(\textbf{x},t)$ describes the underlying physical phenomena modelled by the governing Eq. (1) in space $\textbf{x}$ and time *t*. The forcing term is defined as $f(\textbf{x},t)$, with initial conditions (IC) $g(\textbf{x})$ defined at $t=0$, and boundary conditions (BC) $h(\textbf{x},t)$ defined over the domain boundaries $\partial \Omega$. NNs can map complex and nonlinear features between inputs and outputs through a combination of hidden layers for different network structures and activation functions^36^. In a fully connected NN with $\ell$ hidden layers, the output of the $i^{\text {th}}$ layer (and the input to the $(i+1)^{\text {th}}$ layer) is written as

2 $$\begin{aligned} \boldsymbol{u_{(i)NN}} = \sigma _i (\Theta _i;\boldsymbol{u}_{(i-1)}), \ \ i = 1,2,3,\dots ,\ell +1, \end{aligned}$$

where $\boldsymbol{u_0 = x}$ is the input layer, $\Theta$ represents the weights matrix and biases vector of the $i^{th}$ layer and are referred to as *network parameters* for simplicity, $\sigma _i$ are the activation functions of the layer $\ell$, and $\boldsymbol{u_{(\ell +1)NN}}$ is the NN output. In the remainder of this paper, the NN output is denoted as $u_{NN}$ for simplicity. Hence, the NN output is sought as a NN–parameterized trial function such as

3 $$\begin{aligned} u_{NN}(\textbf{x},t) = NN(\textbf{x},t;\Theta ), \ \ \Theta \in \mathbb {R}^p, \end{aligned}$$

which defines a finite-dimensional nonlinear trial space parameterized by $\Theta$, where *p* denotes the number of trainable parameters.

The solution of Eq. (1) can be approximated by a NN as an optimization problem in which the NN iteratively updates its parameters $\Theta$ to reduce a loss function formulated using the prior knowledge of the PDE. We obtain the Petrov–Galerkin weak-form of the PDE using the same procedure as in Ref^4^ to construct the residual loss function. Starting by moving all PDE terms to the left-hand side, the residual $\mathscr {R}(u_{NN})$ of Eq. (1) is defined as

4 $$\begin{aligned} \mathscr {R}(u_{NN}) = L^{\textbf{q}}[u_{NN}(\textbf{x},t)] - f (\textbf{x},t), \end{aligned}$$

The Petrov–Galerkin method is defined as a weighted residual method in which the trial function and the test function belong to different function spaces^4,37^. Analogously to FEM, we derive the weak-form within the Petrov–Galerkin framework where the nonlinear output of the NN shown in Eq. (3) is the trial solution, while the FEM shape functions $\phi _k(t,x),\ k =1,2,\dots K$, represent the test functions where *K* is the number of test functions^3^. Multiplying Eq. (4) by a test function $\phi _k$, integrating over the domain, and performing integration by parts, we obtain the weak-form of the PDE and the boundary term defined over $\partial \Omega$ in the general form

5 $$\begin{aligned} \mathscr {R}(u_{NN}(\textbf{x},t))_{\text {weak-form}} =a(u_{NN}(\textbf{x},t),\phi _k) - L(\phi _k), \end{aligned}$$

where for linear PDEs, $a(u_{NN},\phi _k)$ is a bilinear form involving all the terms that contain the trial and test functions, while $L(\phi _k)$ is a linear form containing all the terms involving only the latter^4,37^. In contrast, for nonlinear PDEs the weak form generally leads to a nonlinear operator acting on $u_{NN}$, which cannot be expressed as a bilinear form. To construct the FENNM residual loss function, the weak-form obtained in Eq. (5) is evaluated for each element *n* inside the domain for each test function $\phi _k$ separately. Hence, the residual loss function is defined for the $n^{\text {th}}$ element and $k^{\text {th}}$ test function $\phi _k$ as follows

6 $$\begin{aligned} \mathscr {L}_\mathscr {R}^{(n,k)} = (a(u_{NN}(\textbf{x}^{(n)},t^{(n)}),\phi _k) - L(\phi _k))^2. \end{aligned}$$

Squaring and summing the residual obtained for each test function over each element yields a positive-definite^10,14,17^, energy-like norm and results in a well-posed least-squares minimization consistent with the weak formulation^38^. The residual loss function for a discretized mesh consisting of $\mathscr {N}_{el}$ elements for *K* test functions becomes

7 $$\begin{aligned} \mathscr {L}_{\mathscr {R}} = \frac{1}{\mathscr {N}_{el}K}\sum ^{\mathscr {N}_{el}}_{n=1} \sum _{k=1}^K(a(u_{NN}(\textbf{x}^{(n)},t^{(n)}),\phi _k) - L(\phi _k))^2. \end{aligned}$$

The total loss function comprises the residual, the BCs, and the ICs loss functions and defined as

8a $$\begin{aligned} \mathscr {L}&= \tau _\mathscr {R}\mathscr {L}_\mathscr {R} + \tau _\mathscr {B}\mathscr {L}_\mathscr {B} + \tau _\mathscr {I}\mathscr {L}_\mathscr {I}, \end{aligned}$$

8b $$\begin{aligned} \mathscr {L}_\mathscr {B}&= \frac{1}{\mathscr {N}_{\mathscr {B}}} \sum _{i=1}^{\mathscr {N}_{\mathscr {B}}}(u(\textbf{x}_i,t_i) - h(\textbf{x}_i,t_i))^2, \end{aligned}$$

8c $$\begin{aligned} \mathscr {L}_\mathscr {I}&= \frac{1}{\mathscr {N}_{\mathscr {I}}} \sum _{i=1}^{\mathscr {N}_{\mathscr {I}}}\bigg (u(\textbf{x}_i,t_i)\bigg |_{t_i=0} - g(\textbf{x}_i)\bigg )^2, \end{aligned}$$

where the penalty terms $\tau _{\mathscr {R}}$, $\tau _{\mathscr {B}}$, and $\tau _{\mathscr {I}}$ represent loss weight parameters for the residual loss term $\mathscr {L}_{\mathscr {R}}$, the BCs loss term $\mathscr {L}_{\mathscr {B}}$, and the ICs loss term $\mathscr {L}_{\mathscr {I}}$, respectively.

### Implementation of the FENNM loss function

We use the one-dimensional Burgers’ equation as an illustrative example to explain the construction of the total loss function introduced in Sect. Construction of the FENNM loss function. This example also serves as a reference case to facilitate the reproduction of the results. Owing to its wide range of applications across different domains^39^, it is later revisited as a case study in Sect.  One-dimensional Burgers’ equation. The equation takes the form

9 $$\begin{aligned} \frac{\partial u}{\partial t} + u \frac{\partial u}{\partial x} = \nu \frac{\partial ^2 u}{\partial x^2}, \end{aligned}$$

over the domain $(t,x) \in (0,1]\times [-1,1]$, *u* is the velocity, and $\nu$ is the kinematic viscosity. The BCs and ICs are defined, respectively as

10a $$\begin{aligned} u(t, x)\bigg |_{x=-1}&= u(t, x)\bigg |_{x=1} = 0, \end{aligned}$$

10b $$\begin{aligned} u(t, x)\bigg |_{t=0}&= -\sin (\pi x). \end{aligned}$$

We get the residual $\mathscr {R}(u_{NN})$ of Eq. (9) by moving all the terms to the left-hand side as

11 $$\begin{aligned} \mathscr {R}(u_{NN}) = \frac{\partial u_{NN}}{\partial t} + u_{NN} \frac{\partial u_{NN}}{\partial x} - \nu \frac{\partial ^2 u_{NN}}{\partial x^2}. \end{aligned}$$

The residual loss function defined for the $n^{\text {th}}$ element and $k^{\text {th}}$ test function $\phi _k$ becomes

12 $$\begin{aligned} \begin{aligned}&\mathscr {L}_\mathscr {R}^{(n,k)} = \bigg ( \int _{t^{(n)}} \int _{x^{(n)}} \phi _k(t,x) \left( \frac{\partial u_{NN}}{\partial t} + u_{NN} \frac{\partial u_{NN}}{\partial x} \right) dx \, dt \\&+ \int _{t^{(n)}} \int _{x^{(n)}} \frac{\partial \phi _k(t,x)}{\partial x} \left( \nu \frac{\partial u_{NN}}{\partial x} \right) dx \, dt - \nu \int _{t^{(n)}} \left. \phi _k(t,x) \frac{\partial u_{NN}}{\partial x} \right| _{\partial \Omega _{x^{(n)}}} dt \bigg )^2, \end{aligned} \end{aligned}$$

The residual loss function for a discretized mesh consisting of $\mathscr {N}_{el}$ elements for *K* test functions is given by

13 $$\begin{aligned} \begin{aligned}&\mathscr {L}_\mathscr {R}=\frac{1}{\mathscr {N}_{el}K} \sum _{n=1}^{\mathscr {N}_{el}}\sum _{k=1}^K \bigg ( \int _{t^{(n)}} \int _{x^{(n)}} \phi _k(t,x) \left( \frac{\partial u_{NN}}{\partial t} + u_{NN} \frac{\partial u_{NN}}{\partial x} \right) dx \, dt \\&+ \int _{t^{(n)}} \int _{x^{(n)}} \frac{\partial \phi _k(t,x)}{\partial x} \left( \nu \frac{\partial u_{NN}}{\partial x} \right) dx \, dt - \nu \int _{t^{(n)}} \left. \phi _k(t,x) \frac{\partial u_{NN}}{\partial x} \right| _{\partial \Omega _{x^{(n)}}} dt \bigg )^2. \end{aligned} \end{aligned}$$

However, the integral form in Eq. (13) cannot be defined directly within the NN and has to be approximated using numerical integration techniques such as the Gauss quadrature rule^10^. Consequently, the domain must be transformed from the global coordinates (*t*, *x*) to the local coordinates $(\tau ,\xi ) = [-1,1]\times [-1,1]$. The residual loss function $\mathscr {L}_\mathscr {R}$ becomes

14 $$\begin{aligned} {\begin{aligned} \mathscr {L}_\mathscr {R}&= \frac{1}{\mathscr {N}_{el}K} \sum _{n=1}^{\mathscr {N}_{el}} \sum _{k=1}^{K} \Bigg ( \sum _{q=1}^{Q} W_q \, \hat{\phi }_k(\tau _q,\xi _q) \Bigg ( \frac{\partial u_{NN}(t_q^{(n)},x_q^{(n)})}{\partial t} + u_{NN}(t_q^{(n)},x_q^{(n)}) \, \frac{\partial u_{NN}(t_q^{(n)},x_q^{(n)})}{\partial x} \Bigg ) J_x J_t \\&\quad + \sum _{q=1}^{Q} W_q \, \frac{\partial \hat{\phi }_k(\tau _q,\xi _q)}{\partial x} \left( \nu \, \frac{\partial u_{NN}(t_q^{(n)},x_q^{(n)})}{\partial x} \right) J_x J_t \\&\quad - \sum _{q=1}^{Q_t} \nu \, W_q \left. \hat{\phi }_k(\tau _q,\xi _q) \frac{\partial u_{NN}(t_q^{(n)},x_q^{(n)})}{\partial x} \right| _{\partial \Omega _{x^{(n)}}} J_t \Bigg )^2, \end{aligned}} \end{aligned}$$

where the two-dimensional integral is approximated using a number *Q* of quadrature points, while the one-dimensional boundary integral is approximated using a number $Q_t$ of quadrature points, where the subscript *t* refers to the time. The weight $W_q$ of the $q^{\text {th}}$ quadrature in the element is located at the $(t_q, x_q)$ global coordinates. When elements have regular shapes, their Jacobian can be defined as the product of two one-dimensional Jacobians, $J_x \times J_t$. The test functions $\hat{\phi }_k$ are formulated in the local coordinate system, enabling their repeated application across elements of varying sizes. Furthermore, the quadrature weights are fixed for a given Gauss quadrature order. As a result, Eq. (14) comprises a fixed coefficient across all elements within the domain consisting of Gauss quadrature weights multiplied by the test functions $W_q \hat{\phi }_k(\tau _q, \xi _q)$. The PDE operators are global components defined in global coordinates for all elements at the quadrature points. Therefore, the evaluation of the weak-form residual loss function can be performed in parallel for all test functions using convolution operations, accelerating the loss function computations.

The total loss function comprises the residual loss function and the boundary and initial conditions of the PDE as follows

15a $$\begin{aligned} \mathscr {L}&= \tau _\mathscr {R}\mathscr {L}_\mathscr {R} + \tau _\mathscr {B}\mathscr {L}_\mathscr {B} + \tau _\mathscr {I}\mathscr {L}_\mathscr {I}, \end{aligned}$$

15b $$\begin{aligned} \mathscr {L}_\mathscr {B}&= \frac{1}{N_{\mathscr {B}}} \sum _{i=1}^{N_{\mathscr {B}}}\bigg (u_{NN}(t_i,x_i)\bigg |_{x_i=-1}\bigg )^2 + \frac{1}{N_{\mathscr {B}}} \sum _{i=1}^{N_{\mathscr {B}}}\bigg (u_{NN}(t_i,x_i)\bigg |_{x_i=1}\bigg )^2, \end{aligned}$$

15c $$\begin{aligned} \mathscr {L}_\mathscr {I}&= \frac{1}{N_{\mathscr {I}}} \sum _{i=1}^{N_{\mathscr {I}}}\bigg (u_{NN}(t_i,x_i)\bigg |_{t_i=0}+\text {sin}(\pi x_i)\bigg )^2. \end{aligned}$$

The same procedure is used to derive the total loss function for the remaining PDEs in this work.

### Schematic representation of the two-dimensional FENNM training workflow

**Fig. 1:**
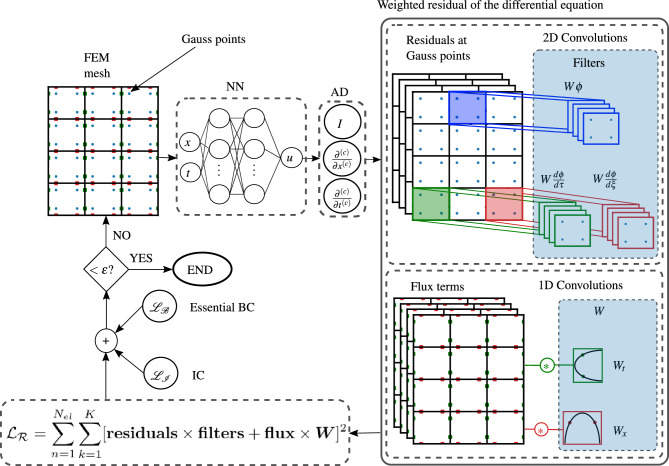
Schematic of two-dimensional FENNM. The NN outputs formulate the differential equation residuals and fluxes. Then predefined filters pass over them to compute the Gauss quadrature sums using two and one-dimensional convolution operations, respectively, before evaluating the global residual loss function.

Figure 1 shows a schematic representation of the two-dimensional FENNM, where the NN generates the PDE operators that are used to construct the weak-form weighted residual and element fluxes using AD. The filters consist of the Gauss quadrature weights and the test functions with their corresponding partial derivatives. Then, two-dimensional convolution operations are applied to the weak-form residuals to construct the integral part of the loss function.

The elements’ boundary terms consist of fluxes, test functions, and Gauss quadrature weights. They are evaluated via one-dimensional convolution operations, where the Gauss quadrature weights serve as a one-dimensional filter that passes over the signals obtained by multiplying the fluxes with the test functions. These signals are equivalent to the number of test functions used in the weak formulation. After that, the outputs of the convolution operations are summed to construct the complete residual loss function map. Finally, the loss terms are multiplied by their corresponding penalty parameters and summed to construct the total loss function.

Hence, the terms in Eq. (14) are grouped as follows

16 $$\begin{aligned} \begin{aligned} \mathscr {L}_\mathscr {R}&= \frac{1}{\mathscr {N}_{el}K}\sum _{n=1}^{\mathscr {N}_{el}} \sum _{k=1}^K \Bigg (\frac{1}{J_x J_t}\Bigg ( \sum _{q=1}^Q \underbrace{W_q \phi _k(\tau _q,\xi _q)}_{\text {Filters 1}} \underbrace{\left( \frac{\partial u_{NN}(t_q^{(n)},x_q^{(n)})}{\partial t} + u_{NN}(t_q^{(n)},x_q^{(n)}) \frac{\partial u_{NN}(t_q^{(n)},x_q^{(n)})}{\partial x} \right) }_{\text {PDE operators}} J_x \, J_t \\&+ \sum _{q=1}^Q \underbrace{W_q \frac{\partial \phi _k(\tau _q,\xi _q)}{\partial x}}_{\text {Filters 2}} \underbrace{\left( \nu \frac{\partial u_{NN}(t_q^{(n)},x_q^{(n)})}{\partial x} \right) }_{\text {PDE operators}} J_x \, J_t - \sum _{q=1}^{Q_t} \underbrace{W_q}_{\text {Filter 3}} \underbrace{\left. \nu \phi _k(\tau _q,\xi _q) \frac{\partial u_{NN}(t_q^{(n)},x_q^{(n)})}{\partial x} \right| _{\partial \Omega _{x^{(n)}}}}_{\text {Fluxes}} J_t \Bigg )\Bigg )^2, \end{aligned} \end{aligned}$$

where we normalize the elements by their corresponding Jacobians because the residual loss depends on the mesh size and, consequently, on the Jacobian values, which can reduce its contribution to the total loss function if the elements are small.

Filters 1 are two-dimensional filters consisting of the test functions and the Gauss quadrature weights, applied to the two-dimensional PDE operators that are not integrated by parts. Filters 2 represent two-dimensional filters consisting of the partial derivative of the test functions and the Gauss quadrature weights, applied to the two-dimensional PDE operators that are integrated by parts in the spatial coordinates. One-dimensional convolution is used to evaluate the boundary terms of each element, where Filter 3 represents the Gauss quadrature weights, and the fluxes comprise the test functions and PDE operators evaluated at the element edges in the temporal domain. Figure 12 in Appendix "An illustration of FENNM meshes and two-dimensional convolution filters" illustrates a two-dimensional $4\times 3$ uniform mesh with four quadrature points per element using the weighted residual in strong-form (a), and when using the weighted residual in weak-form (b). Figure 13 in Appendix "An illustration of FENNM meshes and two-dimensional convolution filters" illustrates the bilinear filters generated using Lagrange linear test functions for regular quadrilateral elements.

For all case studies, unless specified otherwise, the penalty terms $\tau _{\mathscr {R}}$, $\tau _{\mathscr {B}}$, and $\tau _{\mathscr {I}}$ are defined as nondecreasing variables and are simultaneously updated during the training process using the ADAM optimizer^40,41^ and remain unchanged when switching to the L-BFGS optimizer^42^. Consequently, loss terms with increasing errors are assigned higher weights, forcing the NN to minimize them. Hence, the optimizers search for a saddle point that reduces the total loss function using gradient descent and gradient ascent to update the penalty terms. The rate of convergence of FENNM, its dependence on the order of the test functions and the Gauss quadrature rule, and its comparison with FEM are discussed in ref^26^.

## Case studies

FENNM explicitly includes the flux terms within the residual loss function. In this section, we demonstrate the advantages and benefits that are derived by using nonvanishing FEM shape functions as a test function. In addition, we show how we extend the FENNM formulation to two-dimensional domains, where the second dimension can be space, time, or a parameter.

We present several case studies to illustrate the application of FENNM to a variety of problems in fluid mechanics and heat transfer. The examples cover both the one-dimensional and the steady-state one-dimensional Burgers equations, the lid-driven cavity flow problem, the thermal behavior prediction of a simplified satellite panel, and the heat transfer for a two-dimensional section of a turbine blade. Table 1 presents the NN architectures, associated parameters, and selected test functions used in the case studies presented in Sects. "One-dimensional Burgers’ equation" through "Heat transfer in a turbine blade" to ensure reproducible results.

**Table 1:** FENNM design parameters for the case studies presented in Sects. One-dimensional Burgers’ equation through "Heat transfer in a turbine blade".

Section	Case study	Number of neurons/layer	Number of layers	Activation function	Number of elements	Test functions	Quadrature points/element	Epochs	
		$\mathscr {N}$	$\ell$	$\sigma$	$\mathscr {N}_{el}$	$\phi$	*Q*	ADAM/ Learning rate	L-BFGS
One-dimensional Burgers’ equation	1D Burgers	20	4	tanh	$23 \times 18$	Lagrange Linear	$3 \times 3$	7500 $1 \times 10^{-4}$	12500
Parameter analysis for one-dimensional steady-state Burgers’ equation	Steady-state 1D Burgers	20	4	sin	$10\times 11$	Serendipity Quadratic	$7 \times 7$	5000 $5 \times 10^{-4}$ 5000 $1 \times 10^{-4}$ 10000 $5 \times 10^{-5}$	20000
Lid-driven cavity flow	Lid-driven cavity flow	20	4	tanh	$121\times 121$	Lagrange Quadratic	$5\times 5$	10000 $1 \times 10^{-4}$	880000
Satellite panel	Satellite panel	20	2	tanh	$20\times 20$	Lagrange Linear	$4\times 4$	5000 $1 \times 10^{-4}$	10000
Heat transfer in a turbine blade	Turbine blade	40	4	tanh	1062	Lagrange Cubic	$5\times 5$	1000 $1 \times 10^{-3}$	90000

### One-dimensional Burgers’ equation

We consider the one-dimensional Burgers’ equation (9), which represents a benchmark problem for testing numerical methods as it can lead to solutions with a steep velocity gradient that morphs into a shock for small values of viscosity $\nu<<1$^43^. Moreover, it is a simplification of the Navier-Stokes equations, where physics becomes more complex as the nonlinear term dominates, requiring robust numerical methods to accurately capture the flow properties^44^. This initial boundary value problem allows us to examine the effect of local mesh refinement on approximation accuracy.

**Fig. 2:**
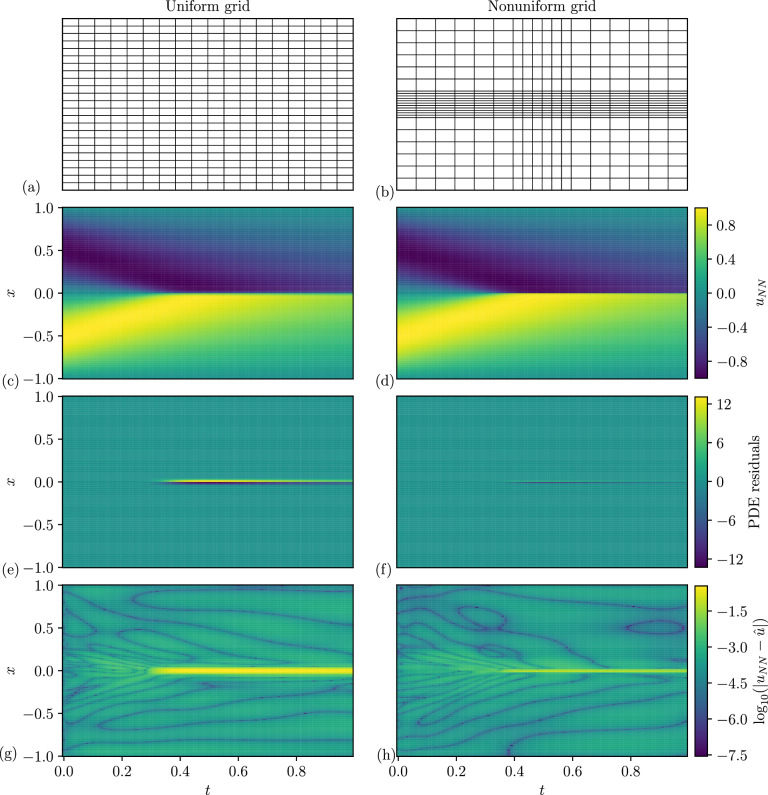
The solution of one-dimensional Burgers’ equation using FENNM: (**a** and **b**) uniform and nonuniform meshes of $23 \times 18$ elements, respectively; (**c** and **d**) FENNM prediction for uniform and nonuniform meshes, respectively; (**e** and **f**) Strong-form PDE residuals for uniform and nonuniform meshes, respectively; (**g** and **h**) PWE for uniform and nonuniform meshes, respectively.

Figure 2 presents the solution to the one-dimensional Burgers’ equation using FENNM on both uniform and nonuniform grids. Furthermore, it shows the associated PDE residuals and the absolute point-wise error (PWE) evaluated as the logarithm of the absolute error, $log_{10}(|u_{NN}-\hat{u}|)$, where $\hat{u}$ is the solution taken from Ref^17^ for a viscosity of $\nu = 0.01/ \pi$. Both meshes consist of 23 elements in space and 18 elements in time, and both cases employ the same Gauss quadrature rule, test functions, network architecture, and hyperparameters listed in Table 1.

The left-hand side of Fig. 2 shows the solution to Burgers’ equation on a uniform mesh, shown in (a). Figure 2(c) presents the FENNM approximation, which captures the general behavior of the solution in the domain. However, the PDE residuals shown in Fig. 2(e), which are not included in the training process and computed *a posteriori* by the trained NN, exhibit high values at the location of discontinuity. This is correlated with a high PWE compared to the exact solution of^17^ as demonstrated in Fig. 2(g). The PDE strong-form residual can be computed with the trained FENNM and acts as a proxy for the PWE, which is inaccessible in a real application case.

On the right-hand side of Fig. 2, local mesh refinement is applied in the computational domain based on the observed residual of Fig. 2(e) using the same number of elements. A coarser mesh is used in regions with small residuals, while a denser mesh is applied at locations of high residuals, as demonstrated in Fig. 2(b). As expected, the FENNM approximation shown in Fig. 2(d) reduces the PDE residuals in the discontinuity regions as presented in Fig. 2(f). Consequently, the PWE is reduced in the same regions as illustrated in Fig. 2(h).

**Fig. 3:**
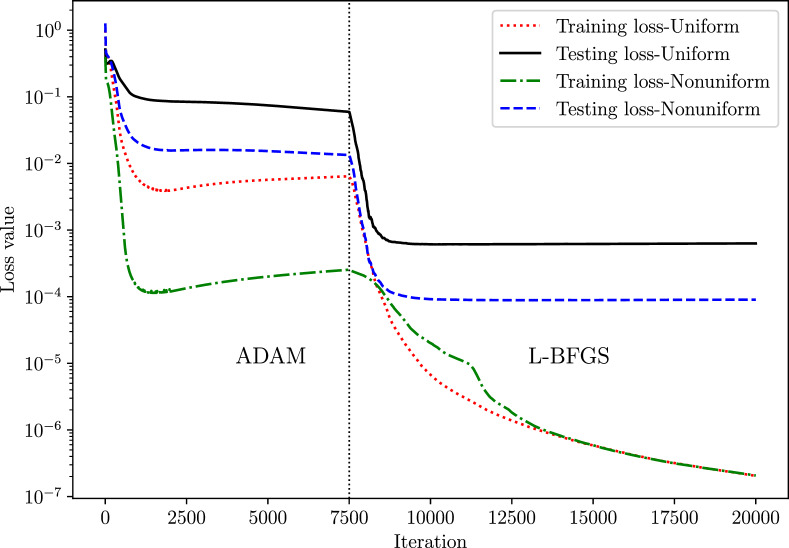
Average training and testing histories of FENNM over ten runs with random initialization for the one-dimensional Burgers’ equation using uniform and nonuniform meshes.

Figure 3 shows the average training and testing histories, measured by mean squared error (MSE), over ten runs with random initialization for the one-dimensional unsteady Burgers’ equation using FENNM on uniform and nonuniform meshes. We observe that FENNM reaches the same training residual value toward the end of training for both meshes. However, the residual value alone does not indicate which mesh yields better results. The testing losses – which are calculated using the reference solution provided in Ref^17^ – show that local mesh refinement improves the accuracy of FENNM even when using the same number of elements. A key challenge is that we do not always have access to exact or experimental data for comparison. In such cases, the strong-form residual provides valuable insight to guide local mesh refinement as shown in Fig. 2. This residual is not included during training and can be computed at no additional data cost, as it only requires an extra backpropagation step. In contrast, PINNs rely on the strong-form residual during training; therefore, it cannot be used independently to guide local mesh refinement, as explained in Sect. "Parameter analysis for one-dimensional steady-state Burgers’ equation".

### Parameter analysis for one-dimensional steady-state Burgers’ equation

Refining a given parameter using traditional FEM solvers requires an iterative procedure, performing multiple simulations with different parameter values. Additionally, each simulation requires considerable storage space. Alternatively, FENNM can be extended for design problems and parameter optimization tasks. The parameter of interest is incorporated into the FENNM framework as an extra input, and then the model is trained over a specified range of that parameter. Furthermore, FENNM offers a continuous approximation of the solution across the parameter spectrum once trained. The storage requirements for NN parameters in general range from just a few kilobytes in the case of small models to megabytes for models containing millions of parameters, allowing for easy access to support real-time simulation with specific parameter values.

Consider the following steady state one-dimensional Burgers’ equation with sinusoidal Dirichlet boundary conditions with wavenumber $\kappa$

17a $$\begin{aligned} u\frac{\partial u}{\partial x}- \frac{\partial ^2u}{\partial x^2}&= f(\kappa ,x), \end{aligned}$$

17b $$\begin{aligned} u(\kappa ,x)\bigg |_{x=-1} = \sin (-\kappa ),&\quad u(\kappa ,x)\bigg |_{x=1} = \sin (\kappa ), \end{aligned}$$

where $(\kappa , x) \in [-\pi ,10\pi ]\times [-1,1]$. A manufactured analytical solution is given by

18 $$\begin{aligned} u(\kappa ,x) = sin(\kappa x), \end{aligned}$$

where the forcing term $f(\kappa ,x)$ is obtained by substituting the analytical solution (18) into Eq. (17).

It is possible to solve Eq. (17) for a wavenumber range treating the wavenumber $\kappa$ as an additional dimension in the FENNM formulation. This is accomplished by extending the test functions in the spatial dimension *x* and the wavelength parameter dimension $\kappa$. Hence, the weak-form loss function becomes

19 $$\begin{aligned} \begin{aligned} \mathscr {L}_\mathscr {R}&=\frac{1}{\mathscr {N}_{el}K} \sum _{n=1}^{\mathscr {N}_{el}} \sum _{k=1}^K \Bigg ( \sum _{q=1}^Q W_q \hat{\phi }_k(\hat{\kappa }_q,\xi _q) \left( u_{NN} \frac{\partial u_{NN}}{\partial x} - f(\kappa _q^{(n)},x_q^{(n)}) \right) J_x \, J_\kappa \\&+ \sum _{q=1}^Q W_q \frac{\partial \hat{\phi }_k(\hat{\kappa }_q,\xi _q)}{\partial \xi } \frac{\partial u_{NN}}{\partial x} J_x J_\kappa - \sum _{q=1}^{Q_w} W_q \left. \hat{\phi }_k(\hat{\kappa }_q,\xi _q) \frac{\partial u}{\partial x} \right| _{\partial \Omega _{x^{(n)}}} J_\kappa \Bigg )^2, \end{aligned} \end{aligned}$$

where $\hat{\kappa }$ is the wavenumber in the local coordinates. The problem in Eq. (19) is thus solved in both space $x \in [-1, 1]$ and parameter space $\kappa \in [-\pi , 10\pi ]$ in one training.

**Fig. 4:**
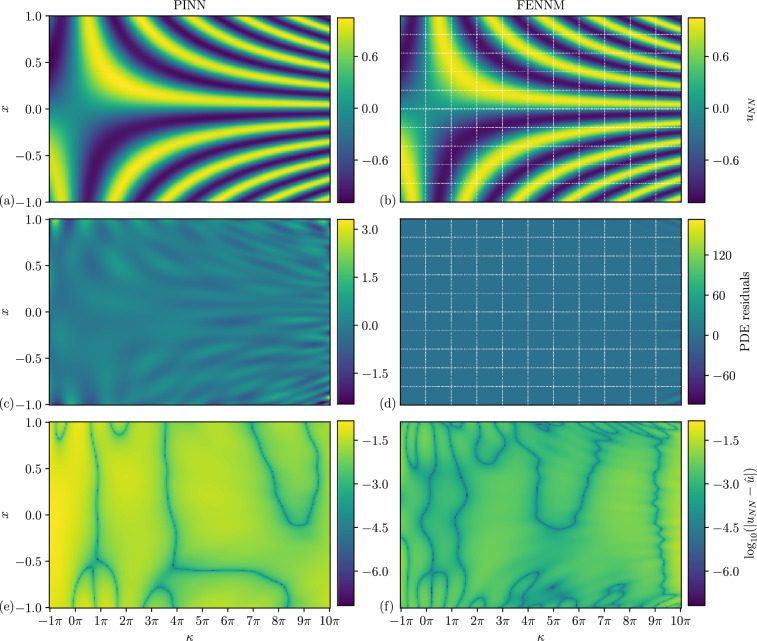
The solution of one-dimensional steady-state Burgers’ equation over the parameter space ($\kappa$); (**a** and **b**) solution approximation by PINN and FENNM, respectively; (**c** and **d**) PDE residuals by PINN and FENNM; (**e** and **f**) PWE by PINN and FENNM, respectively.

Figure 4 shows a comparison between the vanilla PINN and the FENNM approximations for Eq. (17) compared to the analytical solution. Both methods use similar initialization, network architecture, hyperparameter settings, and are trained for the same number of iterations using both optimizers as listed in Table 1. As expected, both the vanilla PINN and the FENNM capture the behavior of the solution within the domain, as illustrated in Fig. 4 (a and b), respectively. However, FENNM exceeds the vanilla PINN in terms of accuracy by one order of magnitude as shown in (e and f), respectively. The left and right sides of the domain correspond to the extremities of the wavelength $\kappa$. The vanilla PINN struggles to capture the solution at these extremities, while FENNM has the highest error at the same locations. We can mitigate these errors by solving for PINN or FENNM at each of these extremities separately and adding their approximation to the total loss as additional data. Moreover, the vanilla PINN struggles to capture the solution when the wavelength number sign changes from negative to positive. In the literature, it has been shown that the vanilla PINN suffers as the wavelength of the solution increases^45^, which is observed on the right side of the domain. On the other hand, FENNM approximates the solution with PWE of order ($\mathscr {O}^{-2}$) throughout the domain with a slight increase in error on the left and right boundaries.

The PDE residuals of the vanilla PINN in Fig. 4(c) are closer to zero compared to the PDE residuals of FENNM, which have higher values in the high wavelength region at the right end of the domain, as shown in Fig. 4(d). PINN uses the strong-form PDE residuals to optimize the NN parameters during training. However, a small residual value in PINN does not guarantee a small error value in the approximation, as illustrated in Fig. 4(e).

We define the $H^1$ norm in Eq. (20) to quantitatively evaluate the error in FENNM and PINN predictions and their gradients compared to the exact solution over the entire domain:

20 $$\begin{aligned} \Vert u - u_{NN} \Vert _{H^1(\Omega )} = \left( \iint _{\Omega } |u - u_{NN}|^2 + \left| \frac{du}{dx} - \frac{du_{NN}}{dx}\right| ^2 \, dx \right) ^{1/2}. \end{aligned}$$

Table 2 reports the $H^1$ norm of FENNM and PINN, considering both PINN with sampling similar to FENNM and PINN with random sampling, for different NN sizes. For each configuration, the mean $H^1$ norm and the corresponding standard deviation are computed from ten independent runs with random initializations. All training conditions are kept identical across the three models to ensure a fair comparison. It is evident that FENNM consistently outperforms PINN across all network sizes. Furthermore, the smaller standard deviations observed for FENNM indicate greater robustness and stability with respect to random initialization compared to both PINN variants. Moreover, for deeper NNs, FENNM achieves higher accuracy than PINN even when PINN employs an additional layer. These results demonstrate that FENNM delivers superior accuracy and exhibits enhanced robustness, stability, and scalability compared to PINN across different network architectures.

**Table 2:** Comparison of $H^1$ norm errors for different NN depths (mean ± standard deviation)...

Method	$\ell = 1$	$\ell = 2$	$\ell = 3$	$\ell = 4$
FENNM	$25.09 \pm 0.59$	$0.111 \pm 0.071$	$0.0599 \pm 0.0107$	$0.0535 \pm 0.0127$
PINN (random)	$44.57 \pm 15.28$	$0.530 \pm 0.557$	$0.0995 \pm 0.0200$	$0.0575 \pm 0.0204$
Improvement over PINN (random)	$43.70\%$	$79.06\%$	$39.86\%$	$7.06\%$
PINN (uniform)	$31.71 \pm 1.82$	$0.550 \pm 0.279$	$0.132 \pm 0.044$	$0.0621 \pm 0.0131$
Improvement over PINN (uniform)	$20.87\%$	$79.82\%$	$54.62\%$	$13.96\%$

### Lid-driven cavity flow

Fluid dynamics within confined domains is prevalent in many engineering contexts, including lubrication systems, aerodynamic designs, and thermal cooling systems^33^, where boundary-driven motion influences the system’s behavior. These phenomena are described by the Navier-Stokes equations.

The lid-driven cavity flow features a flow inside a square cavity in which the top wall moves horizontally at a constant speed, while the other three walls undergo no-slip condition^46^. It is a benchmark problem that has been used for decades to validate new numerical methods^47,48^. This is because despite its simple geometry, it exhibits complex flow features such as primary vortex^46^, secondary vortices^48^, and it has two singularities in the boundary conditions at the top corners^49^. The steady-state problem is modeled using the Navier-Stokes equations in dimensionless form

21a $$\begin{aligned} \nabla ^*\cdot \textbf{u}^{*}&= 0, \end{aligned}$$

21b $$\begin{aligned} \textbf{u}^{*} \cdot \nabla ^*\textbf{u}^{*}&= -\nabla ^*p^{*} + \frac{1}{Re} \nabla ^{*2} \textbf{u}^{*}. \end{aligned}$$

where $\textbf{u}^*$, $p^*$, $\nabla ^*$, and *Re* are the dimensionless velocity vector, pressure, gradient operator, and Reynolds number, respectively. Eq. (21a) is the continuity equation and Eq. (21b) is the momentum equation expressed using the following dimensionless variables

22 $$\begin{aligned} x^*= \frac{x}{L}, \quad y^*= \frac{y}{L}, \quad \textbf{u}^*= \frac{\textbf{u}}{U}, \quad p^*= \frac{p}{\rho U^2}, \quad \nabla ^*= L \nabla , \quad Re = \frac{UL}{\nu }, \end{aligned}$$

where $x^*, y^*$ and *x*, *y* are the dimensionless and dimensional horizontal and vertical coordinates, respectively. The cavity is a square with size $L=1$, filled with a fluid of density $\rho =1$ and kinematic viscosity $\nu =0.01$, driven by the lid moving at $U=1$, such that $Re=100$, for pressure *p*.

**Fig. 5:**
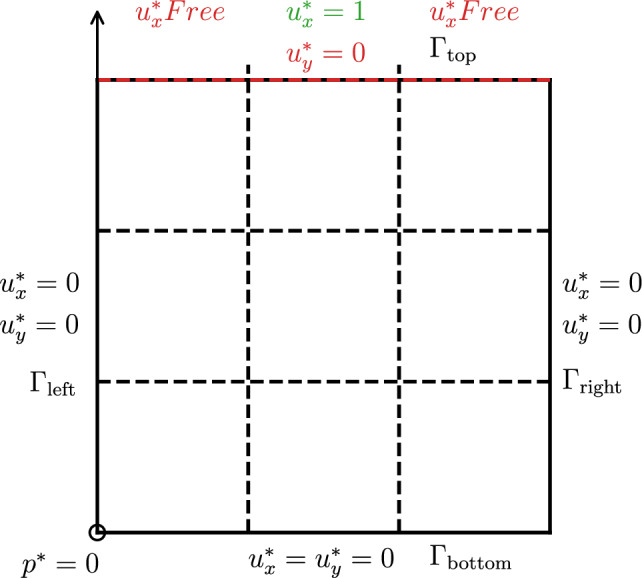
Boundary conditions of the lid-driven cavity where $u_x^*$ is set free at the top corners.

The boundary conditions are

23a $$\begin{aligned} \textbf{u}^*&= \textbf{0} \quad \text {on} \quad \Gamma _{\text {left}} \cup \Gamma _{\text {right}} \cup \Gamma _{\text {bottom}}, \end{aligned}$$

23b $$\begin{aligned} \textbf{u}^*&= (1, 0) \quad \text {on} \quad \Gamma _{\text {top}}, \end{aligned}$$

23c $$\begin{aligned} p^*&\bigg |_{x^*= 0, y^*= 0} = 0 \quad , \end{aligned}$$

where $\Gamma _{\text {left}}$, $\Gamma _{\text {right}}$, $\Gamma _{\text {bottom}}$, and $\Gamma _{\text {top}}$ are the left, right, bottom, and top edges of the square cavity, respectively. The dimensionless pressure $p^*$ is set to zero at the domain’s origin. Figure 5 shows a schematic of the application of boundary conditions in the velocity field, detailing how the horizontal velocity component $u^*_x = 1$ in the middle elements at the top, while its value is free in the top corner elements, thanks to the adaptive capabilities of the NN.

We evaluate the precision of FENNM in approximating the lid-driven cavity flow and contrast its results with the FEM mesh-independent solution produced using the open-source software Lethe^50^. Multiplying Eq. (21a) with scalar shape functions $\phi (y,x)$ and Eq. (21b) with vector-valued shape functions $\boldsymbol{\psi }= (\phi _x, \phi _y)$, the weak-form of Eqs. (21a) and (21b) is

24 $$\begin{aligned} \int _{\Omega } (\nabla ^*\cdot \textbf{u}^*)\phi (y,x) \, d\Omega = 0, \end{aligned}$$

25 $$\begin{aligned} \int _{\Omega } \left[ \textbf{u}^*\cdot \nabla ^*\textbf{u}^*\cdot \boldsymbol{\psi } - p^*\nabla ^*\cdot \boldsymbol{\psi } + \frac{1}{Re} \nabla ^*\textbf{u}^*: \nabla ^*\boldsymbol{\psi } \right] \, d\Omega + \int _{\Gamma } \left[ p^*\textbf{n} \cdot \boldsymbol{\psi } - \frac{1}{Re} \nabla ^*\textbf{u}^*\cdot \textbf{n} \cdot \boldsymbol{\psi } \right] \, d\Gamma = 0. \end{aligned}$$

For brevity, the derivation of the loss function for Eqs. (24) and (25) is provided in Appendix "Derivation of the lid-driven cavity loss function".

**Fig. 6:**
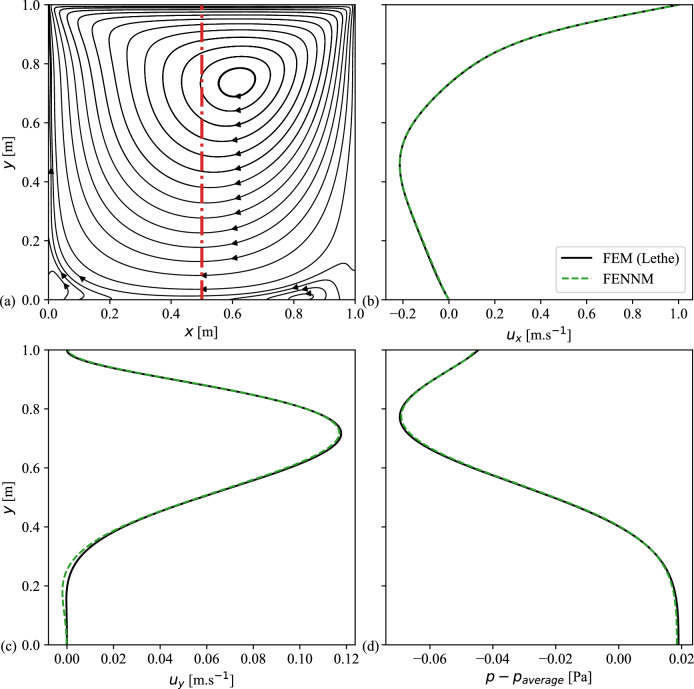
The solution of the lid-driven cavity flow for $Re = 100$; (**a**) Flow streamlines predicted by FENNM; (**b**) Horizontal velocity component at the centerline; (**c**) Vertical velocity component at the centerline; (**d**) Relative pressure at the centerline.

Figure 6 shows a comparison of the FENNM solution using $121\times 121$ elements with the mesh-independent solution obtained by traditional FEM using $1024\times 1024$ elements for the lid-driven cavity flow.

Figure 6(a) shows the flow streamlines predicted by FENNM where it captures the primary vortex in the domain and the secondary vortices in the bottom corners. The centerline velocity components of FENNM and FEM are comparatively illustrated in Fig. 6(b and c). We observe that FENNM captures the velocity near the moving boundary condition similar to FEM. However, the errors are higher in regions where the velocity components are near zero. A comparison between FENNM and FEM in predicting relative pressure $p-p_{average}$ is presented in Fig. 6(d). The overall discrepancies may be due to the different treatment of the top corners’ discontinuities by both approaches.

The consistency observed between FENNM and FEM when applied to the lid-driven cavity flow shows that it can be extended to solve vector-valued PDEs with solutions exhibiting complex features and generate results comparable to those of FEM.

### Satellite panel

PINNs have been extended to heat transfer applications, particularly for predicting, analyzing, and optimizing the thermal efficiency of fins subjected to convective, radiative, and internal heat generation^51–54^. Moreover, they have shown notable effectiveness in addressing inverse problems, especially within one-dimensional contexts^33,55^. Here, we consider the solution of a two-dimensional plate, exemplifying the heat transfer conditions of a satellite panel of area $1\times 1\ \text {m}^{2}$ exposed to solar radiation and connected to a localized heat-generating heat load acting as a finite surface source load. The results are compared with a finite-volume simulation executed using TMG, the advanced thermal solver from Maya HTT^56^. Compared to PINNs, we demonstrate that a localized heat load defined as an area function can be seamlessly incorporated into the FENNM loss function without necessitating special handling in the NN or computational domain.

**Fig. 7:**
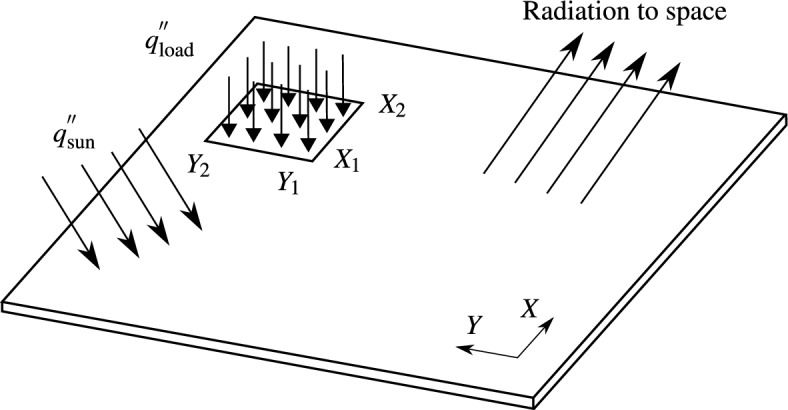
Schematic of satellite panel subjected to solar radiation and a localized heat load.

Figure 7 shows a schematic for the two-dimensional satellite panel subjected to solar radiation and a localized heat load.

The energy balance equation in dimensionless form is

26 $$\begin{aligned} \frac{\partial ^2 T^{*}}{\partial x^{*2}} + \frac{\partial ^2 T^{*}}{\partial y^{*2}} + B_q (q_{\text {sun}}^{*} + q_{\text {load}}^{*}) - B_r \left( (T^{*})^4 - (T_{\text {space}}^{*})^4\right) = 0, \end{aligned}$$

where $T^*$ denotes the dimensionless temperature distribution over the panel. The following dimensionless parameters are used

27 $$\begin{aligned} \begin{aligned} x^{*} = \frac{x}{L}, \quad y^{*} = \frac{y}{L}, \quad T^{*} = \frac{T}{T_0},&\quad T_{\text {space}}^{*} = \frac{T_{\text {space}}}{T_0}, \quad q_{\text {sun}}^{*} = \frac{q^{''}_{\text {sun}}}{q_0}, \quad q_{\text {load}}^{*} = \frac{q^{''}_{\text {load}}}{q_0}, \\ B_q&= \frac{L^2 q_{0}}{k T_{0} d}, \quad B_r = \frac{L^2 \sigma \epsilon T_{0}^3}{k d}, \end{aligned} \end{aligned}$$

for a characteristic length $L = 1\ \text {m}$, characteristic temperature $T_0 = 400\ \text {K}$, and characteristic load $q_0 = 500\ \text {W} \cdot \text {m}^{-2}$. The panel has a thickness of $d= 0.005\ \text {m}$, conductivity $k = 163\ \text {W}\text {m}^{-1}\text {K}^{-1}$, and emissivity $\epsilon = 0.8$. The Stefan--Boltzmann constant is $\sigma = 5.6690 \times 10^{-8} \ \text {W} \cdot \text {m}^{-2} \cdot \text {K}^{-4}$ and the environmental temperature is $T_{\text {space}} = 0\ \text {K}$. To address the steady-state Eq. (26), we assumed that the solar radiation remains constant. The solar radiation and the heat load are defined, respectively

28a $$\begin{aligned} q_{\text {sun}}''&= q_{\text {solar}}'' \alpha \sin \left( \frac{7\pi }{12} \right) , \end{aligned}$$

28b $$\begin{aligned} q_{\text {load}}'' (y,x)&= {\left\{ \begin{array}{ll} 250, & 0.6< x< 0.8, \quad 0.7< y < 0.9, \\ 0, & \text {else}, \end{array}\right. } \end{aligned}$$

where $q_{\text {solar}}'' = 1.377805 \times 10^{3} \ \text {W} \cdot \text {m}^{-2}$ is the solar flux constant and $\alpha = 0.47$ is the absorptivity of the panel. The system is considered adiabatic at all boundaries.

We aim to predict the thermal behavior of the panel in a forward setting, followed by an inverse problem formulation. We demonstrate that FENNM can be used to solve inverse problems, where we identify the unknown absorptivity and the thermal behavior of the panel using one data point.

#### Forward solution

The weak-form loss function of Eq. (26) takes the following form

29 $$\begin{aligned} \begin{aligned} \mathscr {L}_\mathscr {R}&=\frac{1}{\mathscr {N}_{el}K} \sum _{n=1}^{\mathscr {N}_{el}} \sum _{k=1}^K \Bigg ( \sum _{q=1}^Q W_q \frac{\partial \phi _k(\eta _q,\xi _q)}{\partial {\xi }} \frac{\partial {T^*}_{NN}}{\partial {x^*}} J_{{x^*}} J_{{y^*}} - \sum _{q=1}^{Q_{\tilde{y}}} W_q \left. \phi _k(\eta _q,\xi _q) \frac{\partial {T^*}_{NN}}{\partial {x^*}} \right| _{\partial \Omega _{{x^*}^{(n)}}} J_{{y^*}} \\&+ \sum _{q=1}^Q W_q \frac{\partial \phi _k(\eta _q,\xi _q)}{\partial \eta } \frac{\partial {T^*}_{NN}}{\partial {y^*}} J_{{x^*}} J_{{y^*}} - \sum _{q=1}^{Q_{\tilde{x}}} W_q \left. \phi _k(\eta _q,\xi _q) \frac{\partial {T^*}_{NN}}{\partial {y^*}} \right| _{\partial \Omega _{{y^*}^{(n)}}} J_{{x^*}} \\&+ B_q ({q}_{\text {sun}}^*+ {q}_{\text {load}}^*) \sum _{q=1}^Q W_q \phi _k(\eta _q,\xi _q)J_{{x^*}} J_{{y^*}} - B_r \sum _{q=1}^Q W_q \phi _k(\eta _q,\xi _q) ({T}_{NN}^{*4} - {T}_{\text {space}}^{*4}) J_{{x^*}} J_{{y^*}} \Bigg )^2. \end{aligned} \end{aligned}$$

The adiabatic boundary conditions mean that the heat flux at the boundaries is zero. We can impose these boundary conditions inside the residual loss function using nonvanishing FEM shape functions. In other words, there is no competing loss when Neumann or Robin boundary conditions are considered. Hence, the total loss function is reduced to one term provided by Eq. (29), which further improves the NN optimization.

**Fig. 8:**
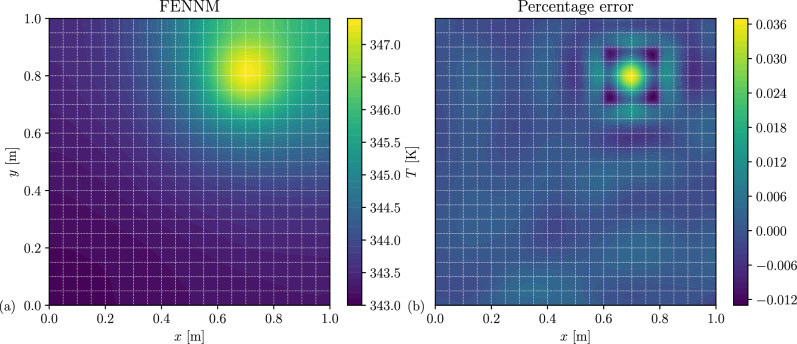
The thermal response of the satellite panel; (**a**) FENNM prediction; (**b**) Percentage error compared to TMG simulation.

Figure 8(a) shows the FENNM solution using $20\times 20$ elements and the percentage error compared to the TMG finite-volume simulation using $100\times 100$ elements in Fig. 8(b) for the heat transfer problem described in Eq. (29).

FENNM predicts the system’s thermal behavior with a $0.036\%$ error using a relatively coarser mesh size. As anticipated, the maximum error is located at the location of the local heat load. This occurs because of the discontinuous nature of the heat load, which subsequently induces sharp changes in the solution within that region. According to Ref^57^, it is observed that PINNs often struggle with abrupt variations and discontinuities in solutions, which can hinder convergence in different areas of the domain. In contrast, FENNM takes advantage of relaxed regularity constraints and the integration of boundary fluxes, enabling the NN to achieve convergence while maintaining precision across the rest of the domain.

#### Inverse solution

In a plausible application scenario, it is reasonable to assume that the panel’s absorptivity of the coatings $\alpha$ degrades over time in a non-trivial manner due to sun exposure. Therefore, our objective is to simultaneously predict its value along with the thermal behavior of the system. In the context of space applications, we often rely on a limited number of sensors, making it beneficial to develop models capable of predicting system unknowns from limited datasets.

To solve for $\alpha$ using FENNM, we define it as a tensor variable with an initial guess in Eq. (28) that is directly substituted in Eq. (29). Then, we define the data loss function using the one data point reading collected from the ideal sensor as follows

30 $$\begin{aligned} \begin{aligned}&{T^*}_{\text {sensor}}(y^*,x^*)\bigg |_{y^* = 0.73,\; x^* = 0.68} = 0.87, \\&\mathscr {L}_{\mathscr {D}} = \Big ( {T^*}_{NN}(y^*,x^*)\bigg |_{y^* = 0.73,\; x^* = 0.68} - {T^*}_{\text {sensor}}(y^*,x^*)\bigg |_{y^* = 0.73,\; x^* = 0.68} \Big )^2. \end{aligned} \end{aligned}$$

Hence, the total loss function becomes

31 $$\begin{aligned} \mathscr {L} = \tau _\mathscr {R}\mathscr {L}_\mathscr {R} +\tau _\mathscr {D}\mathscr {L}_\mathscr {D}. \end{aligned}$$

**Fig. 9:**
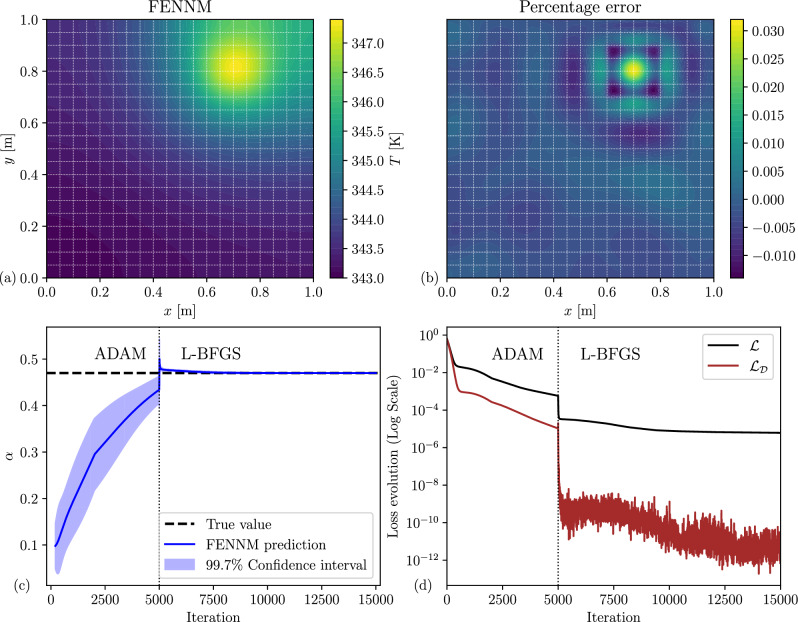
The predicted thermal response of the satellite panel; (**a**) FENNM prediction; (**b**) Percentage error compared to TMG simulation; (**c**) Evolution history of absorptivity $\alpha$; (**d**) Training history.

Figure 9 illustrates the FENNM estimate of the thermal response of the satellite panel along with the predicted absorptivity value $\alpha$.

We performed a stability analysis to verify that FENNM consistently converges to the same solution by running the NN ten times with different initializations. Using the same settings as in Sect. "Satellite panel", Fig. 9(a and b) show the average FENNM estimate of the thermal response of the system and the percentage error, respectively. FENNM can reconstruct the solution throughout the entire domain using one data point for this heat transfer problem. Figure 9(c) shows the average evolution of the prediction $\alpha$ per iteration with a confidence interval of $99.7\%$. The ADAM optimizer has a higher fluctuation than the L-BFGS optimizer. However, ADAM is rapidly approaching the true value of $\alpha$. The average training history illustrated in Fig. 9(d) shows that FENNM reaches convergence around 10,000 iterations and reaches machine precision for the data loss term.

In this case study, we demonstrate the efficacy of FENNM in addressing two-dimensional heat transfer problems subjected to radiation and an external load. The problem is formulated in the weak-form where all the boundary conditions are imposed inside one loss function which is possible due to using the FEM nonvanishing shape functions. Despite the high nonlinearity of the PDE, the nonlinear nature of the trial solution generated by the NN captures the features of the solution using linear shape functions and a relatively coarse mesh. Moreover, FENNM can effectively reconstruct the entire solution using one data point and identify the unknown parameter for the same problem.

### Heat transfer in a turbine blade

Real-world engineering systems rarely exist in idealized or simple domains. Consequently, to advance toward more applicable and industrial use cases, it is essential that the numerical methods developed are capable of handling intricate and irregular geometries. The domain decomposition for irregular geometries in the variational formulation is addressed in FastVPINNs^24,25^, where the weighted residual of the PDE is computed in the weak-form using vanishing test functions. In this section, we demonstrate that FENNM can be seamlessly extended to complex domains using the standard FEM nonvanishing test functions.

**Fig. 10:**
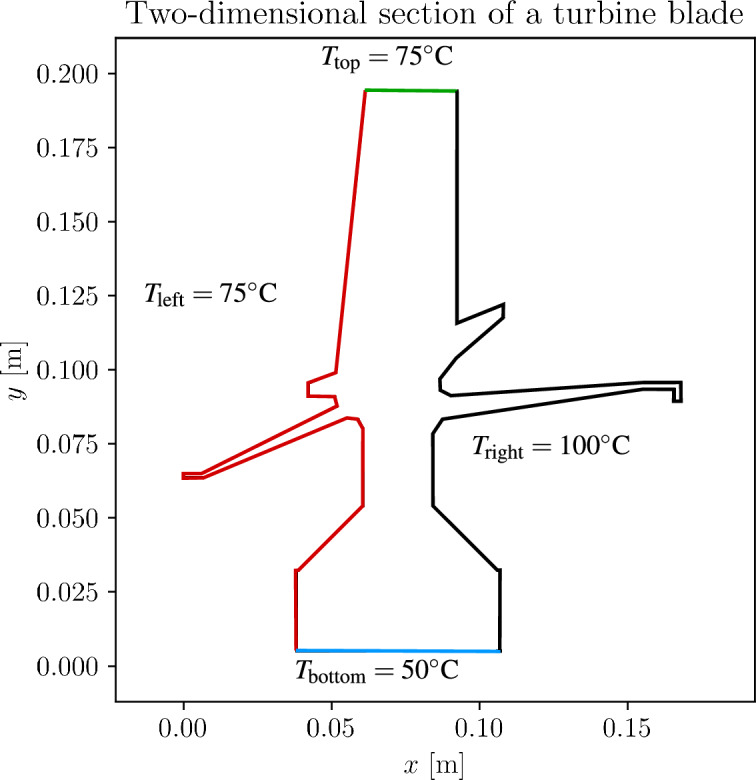
Two-dimensional section of a turbine blade.

Consider the two-dimensional section of a turbine blade shown in Fig. 10. For simplicity of implementation, we consider the turbine blade in a two-dimensional domain. We seek to compute the temperature distribution arising from different temperatures imposed at the boundaries. The heat conduction is governed by the dimensionless Laplace equation

32 $$\begin{aligned} \frac{kT_0}{L^2}\left( \frac{\partial ^2 T^*}{\partial x^{*2}} + \frac{\partial ^2 T^*}{\partial y^{*2}}\right) = 0, \end{aligned}$$

where $k = 6.7\ \text {W}\text {m}^{-1}\text {K}^{-1}$. The temperatures at the boundaries of the body must respect Dirichlet boundary conditions

33 $$\begin{aligned} \begin{aligned}&T_{\text {right}} = 100^\circ \textrm{C}, \\&T_{\text {bottom}} = 50^\circ \textrm{C}, \\&T_{\text {top}} = T_{\text {left}} = 75^\circ \textrm{C}, \end{aligned} \end{aligned}$$

and are imposed with a boundary loss function

34 $$\begin{aligned} \begin{aligned} \mathscr {L}_\mathscr {B}&= \frac{1}{N_{\mathscr {B}\text {-}r}} \sum _{i=1}^{N_{\mathscr {B}\text {-}r}} (T_{NN}^*- T^*_{\text {right}})^2 + \frac{1}{N_{\mathscr {B}\text {-}b}} \sum _{i=1}^{N_{\mathscr {B}\text {-}b}} (T_{NN}^*- T^*_{\text {bottom}})^2 \\&\quad + \frac{1}{N_{\mathscr {B}\text {-}l}} \sum _{i=1}^{N_{\mathscr {B}\text {-}l}} (T_{NN}^*- T^*_{\text {left}})^2 + \frac{1}{N_{\mathscr {B}\text {-}t}} \sum _{i=1}^{N_{\mathscr {B}\text {-}t}} (T_{NN}^*- T^*_{\text {top}})^2. \end{aligned} \end{aligned}$$

The dimensionless form of Eqs. (32) and (33) can be obtained by using similar dimensionless parameters as in Eq. (27), i.e., the temperatures are converted to Kelvin degrees and normalised by $T_0=380\text { K}$. Hence, the strong-form residual loss function becomes

35 $$\begin{aligned} \mathscr {L}_\mathscr {R} = \frac{1}{\mathscr {N}_{el}K}\sum _{n=1}^{\mathscr {N}_{el}} \sum _{k=1}^K \Bigg (\frac{kT_{0}}{L^2} \sum _{q=1}^Q W_q \phi _k(\eta _q,\xi _q) |J|\left( \frac{\partial ^2 T^*_{NN}}{\partial x^{*2}} + \frac{\partial ^2 T^*_{NN}}{\partial y^{*2}} \right) \Bigg )^2, \end{aligned}$$

where |*J*| denotes the determinant of the Jacobian matrix to transfer the coordinates from the global coordinate to the local coordinate.

**Fig. 11:**
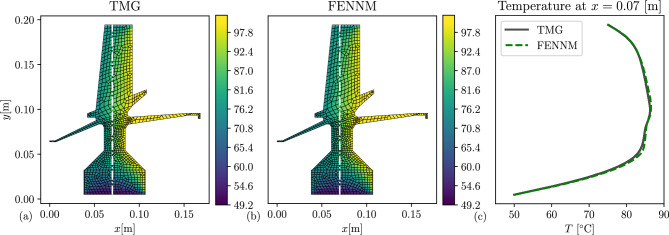
Temperature distribution inside a two-dimensional section of a turbine blade; (**a**) TMG simulation; (**b**) FENNM prediction; (**c**) Temperature variation along the line $x = 0.07 \text { m}$.

**Fig. 12:**
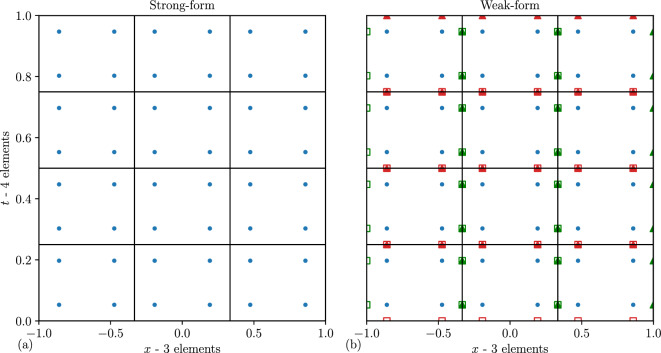
An illustration of a uniform mesh comprising $4 \times 3$ elements over the domain $(t,x) \in [0,1]\times [-1,1]$; (**a**) Computational grid for strong-form weighted residual loss function; (**b**) Computational grid for weak-form weighted residual loss function; Markers indicate:  the quadrature points $(t_q,x_q)$ distributed throughout the elements in the global coordinates;  elements’ lower boundaries;  elements’ upper boundaries;  elements’ left boundaries;  elements’ right boundaries.

**Fig. 13:**
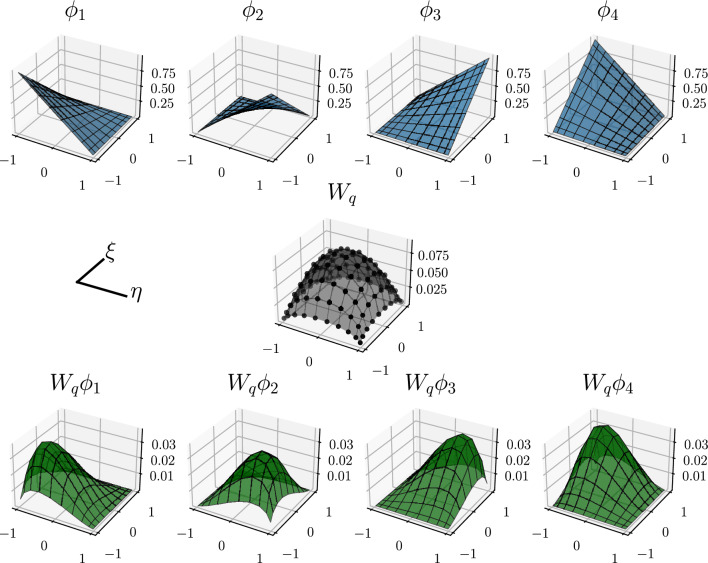
An illustration of the generation of two-dimensional convolution filters using bilinear Lagrange test functions; Top row shows the bilinear Lagrange test functions $\phi _k, k = 1,2,3,4$; Middle row shows Gauss quadrature weights $W_q$; Bottom row shows convolution filters $W_q \phi _k, k = 1,2,3,4$.

Figure 11 illustrates a comparison between the TMG finite-element solution in (a) and the FENNM prediction in (b) for the temperature distribution inside the turbine blade. A single computational mesh was generated using the Gmsh mesh generator^58^ and used for both methods to enable consistent comparison. The FENNM model satisfies the boundary conditions along the outer boundaries of the domain, despite the intricate geometry on the left and right sides. Figure 11(c) shows the temperature variation for the TMG and FENNM solutions along the line $x = 0.07 \text { m}$. The prediction of FENNM satisfies the boundary conditions at the top and bottom of the domain. However, despite a slight deviation in temperature between the two solutions within the interior of the domain, FENNM captures the inflection point similarly to that in the TMG solution. Further convergence studies could be conducted to analyze this discrepancy.

In this section, we illustrate that FENNM is capable of working with irregular meshes generated by tools like Gmsh. We particularly demonstrate the use of convolution operations to compute the weighted residual within the FENNM framework for non-standard geometries, thus opening up possibilities for its adaptation to more complex and real-world scenarios.

## Conclusion

This study highlights the flexibility of the FENNM method by expanding its application to a second dimension, be it spatial, temporal, or parameter space, while incorporating data-driven identification directly into the solution process. The loss function is formulated based on the Petrov–Galerkin framework, where the NN represents the global nonlinear space of trial solution, while the FEM shape functions are used as test functions. Compared to classical FEM, the shape functions in FENNM can be used to derive the weighted residual of the PDEs in the spatial, temporal, and parameter space dimensions. FENNM introduces several key advantages over previous methods:

- In the one-dimensional Burgers’ equation study, FENNM extends the FEM formulation to include spatiotemporal domains and addresses problems that exhibit sharp discontinuities. Using local mesh refinement, FENNM improves the approximation accuracy while providing a continuous solution in time despite the existence of a shock in the solution.
- Similarly to FEM, there is a consistent correlation between the strong-form PDE residuals and the accuracy of FENNM. These residuals, which are not incorporated during training, provide an a posteriori measure that serves as a proxy for local mesh refinement. It is possible to extend local mesh refinement to adaptive mesh refinement, since the properties of the computational mesh, such as the number of elements and quadrature points, are independent of the FENNM model. As a result, different meshes can be integrated smoothly into the training process, while real time evaluation of strong form residuals during training can be used to guide mesh adaptation.
- The FENNM formulation can treat system parameters such as the wavenumber $\kappa$ in the steady state one-dimensional Burgers’ equation as additional dimensions and includes them in the FEM formulation. Hence, FENNM solves for a range of these parameters, creating a library of solutions for design and parameter optimization. This opens future possibilities for using FENNM as a surrogate model in high-dimensional scenarios.
- Solving vector-valued PDEs in fluid mechanics demonstrates FENNM’s ability to maintain fundamental physical properties such as mass conservation and momentum balance and capture complex flow features. Moreover, it highlights potential future applications in systems with fluid-solid interaction.
- The simplified satellite panel problem demonstrates another domain of applications for FENNM. Even when there are high nonlinearities in the system, the nonlinear nature of the FENNM trial function enables it to capture solution features using a relatively coarser mesh than the classical finite-volume method.
- Using one data point to solve for the absorptivity $\alpha$ indicates the ability of FENNM to incorporate data to perform parameter identification to create digital twins. In addition, it suggests that the use of the FEM formulation in the loss function imposes additional constraints that provide strong regularization, reducing the dependence on excessive data.
- FENNM can function effectively on irregular meshes generated by tools such as Gmsh. Using convolution operations to evaluate the weighted residuals within irregular geometries, FENNM demonstrates significant promise for adaptation to more intricate and applicable real-world scenarios.

Future advancements of FENNM will include expanding the framework to three-dimensional or even N-dimensional environments to include the three spatial coordinates, the temporal coordinate, and system parameters for unstructured meshes. In addition, future work may investigate the incorporation of discontinuous Galerkin methods within the FENNM framework for problems involving sharp gradients or discontinuities. The long term goal is to be able to cover model complexity similar to FEM, combined with the possibilities given by the NN, such as parameter exploration and identification.

It is worth noting that, similar to many PINN-based approaches, the computational cost of the proposed method remains higher than that of highly optimized FEM solvers, especially for forward problems. In practice, FENNM as a research-oriented application can be $\mathscr {O}(10)$, $\mathscr {O}(100)$, or even $\mathscr {O}(1000)$ times slower than commercial FEM solvers, depending on the specific implementation. This performance gap is strongly influenced by factors such as the underlying hardware and the programming language used. However, the primary objective of this work is to explore a formulation that bridges concepts between PINN and FEM, rather than to compete with FEM in terms of computational efficiency.

## Data Availability

The datasets generated during and/or analyzed during the current study are available from the corresponding author on reasonable request. The code used to analyze the data is not publicly available due to restrictions from industrial partners but may be available from the corresponding author on reasonable request and with permission of the partners.

## Appendices

### An illustration of FENNM meshes and two-dimensional convolution filters

Figure 12 shows the distribution of the quadrature points per element that FENNM uses to compute the PDE residuals. When using the strong-form weighted residual, only the quadrature points within the elements are required to compute the PDE residuals as depicted in Fig. 12(a). The flux terms appear when the weak-form weighted residual is derived, and FENNM evaluates these fluxes at the edges of the elements, at the same locations as the quadrature points, as shown in Fig. 12(b).

Figure 13 provides a graphical representation of the bilinear Lagrange test functions, the Gauss quadrature weights, and the resulting bilinear convolution filters obtained from their multiplication. The bilinear Lagrange test functions maintain at least a non-vanishing value at each vertex, capturing the flux information between elements when employing the weak-form weighted residual approach. This is significant as it allows for the incorporation of natural boundary conditions within the loss function, thus connecting FENNM to traditional FEM solvers.

### Derivation of the lid-driven cavity loss function

We expand Eqs. (24 and 25) into separate equations as the following:

- The weak-form of the continuity equation
  36 $$\begin{aligned} \int _{y_{\min }^*}^{y_{\max }^*} \int _{x_{\min }^*}^{x_{\max }^*}\left( \frac{\partial u_x^*}{\partial x^*}+\frac{\partial u_y^*}{\partial y^*}\right) \phi J_{x^*} J_{y^*} d x^* d y^*=0 , \end{aligned}$$
  where $u_x$ and $u_y$ denote the components of the velocity vector $\textbf{u}$.
- The weak-form of the momentum equation in the $x^*-$direction
  37 $$\begin{aligned} \begin{aligned}&\int _{y_{\min }^*}^{y_{\max }^*} \int _{x_{\min }^*}^{x_{\max }^*}\left[ \left( u_x^* \frac{\partial u_x^*}{\partial x^*}+u_y^* \frac{\partial u_x^*}{\partial y^*}\right) \psi _x - \frac{1}{J_{x^*}} p^{*} \frac{\partial \psi _x}{\partial x^*} \right. \\&\quad + \left. \frac{1}{Re}\left( \frac{1}{J_{x^*}} \frac{\partial u_x^*}{\partial x^*} \frac{\partial \psi _x}{\partial x^*} + \frac{1}{J_{y^*}} \frac{\partial u_x^*}{\partial y^*} \frac{\partial \psi _x}{\partial y^*} \right) \right] J_{x^*} J_{y^*} \, dx^* \, dy^* \\&+ \int _{y_{\min }^*}^{y_{\max }^*} \left[ \left( p^* - \frac{1}{Re} \frac{\partial u_x^*}{\partial x^*}\right) \psi _x \right] _{x_{\min }^*}^{x_{\max }^*} J_{y^*} \, dy^* \\&- \int _{x_{\min }^*}^{x_{\max }^*} \left[ \frac{1}{Re} \frac{\partial u_x^*}{\partial y^*} \psi _x \right] _{y_{\min }^*}^{y_{\max }^*} J_{x^*} \, dx^* = 0. \end{aligned} \end{aligned}$$
- The weak-form of the momentum equation in the $y^*-$direction
  38 $$\begin{aligned} \begin{aligned}&\int _{y_{\min }^*}^{y_{\max }^*} \int _{x_{\min }^*}^{x_{\max }^*}\left[ \left( u_x^* \frac{\partial u_y^*}{\partial x^*}+u_y^* \frac{\partial u_y^*}{\partial y^*}\right) \psi _y - \frac{1}{J_{y^*}} p^{*} \frac{\partial \psi _y}{\partial y^*} \right. \\&\quad + \left. \frac{1}{Re}\left( \frac{1}{J_{x^*}} \frac{\partial u_y^*}{\partial x^*} \frac{\partial \psi _y}{\partial x^*} + \frac{1}{J_{y^*}} \frac{\partial u_y^*}{\partial y^*} \frac{\partial \psi _y}{\partial y^*} \right) \right] J_{x^*} J_{y^*} \, dx^* \, dy^* \\&+ \int _{x_{\min }^*}^{x_{\max }^*} \left[ \left( p^* - \frac{1}{Re} \frac{\partial u_y^*}{\partial y^*}\right) \psi _y \right] _{y_{\min }^*}^{y_{\max }^*} J_{x^*} \, dx^* \\&- \int _{y_{\min }^*}^{y_{\max }^*} \left[ \frac{1}{Re} \frac{\partial u_y^*}{\partial x^*} \psi _y \right] _{x_{\min }^*}^{x_{\max }^*} J_{y^*} \, dy^* = 0. \end{aligned} \end{aligned}$$

The residual loss function is the addition of the Gauss approximation of the continuity equation loss function and the momentum equation projected on $x^*$ and $y^*$ loss functions for all shape functions as follows:

- The continuity equation loss function
  39 $$\begin{aligned} \mathscr {L}_C= \frac{1}{\mathscr {N}_{el}K} \sum _{n=1}^{N_{el}} \sum _{k=1}^K\left( \frac{1}{J_{x^*}J_{y^*}}\left( \sum _{q=1}^{Q} W_q \phi _k\left( \frac{\partial u_x^*}{\partial x^*}+\frac{\partial u_y^*}{\partial y^*}\right) J_{x^*} J_{y^*}\right) \right) ^2. \end{aligned}$$
- The momentum equation in the $x^*-$direction loss function
  40 $$\begin{aligned} { \displaystyle \begin{aligned} \mathscr {L}_{M, x}&= \frac{1}{\mathscr {N}_{el}K} \sum _{n=1}^{N_{el}} \sum _{k=1}^K \left( \frac{1}{J_{x^*}J_{y^*}}\left( \begin{aligned}&\sum _{q=1}^{Q} W_q \Bigg [ \Big ( u_x^* \frac{\partial u_x^*}{\partial x^*} + u_y^* \frac{\partial u_x^*}{\partial y^*} \Big ) \psi _{x, k} \\&- \frac{1}{J_{x^*}} p^* \frac{\partial \psi _{x, k}}{\partial x^*} + \frac{1}{Re} \left( \frac{1}{J_x} \frac{\partial u_x^*}{\partial x^*} \frac{\partial \psi _{x, k}}{\partial x^*} + \frac{1}{J_y^*} \frac{\partial u_x^*}{\partial y^*} \frac{\partial \psi _{x, k}}{\partial y^*} \right) \Bigg ] J_{x^*} J_{y^*} \\&+ \sum _{q=1}^{Q_{y^*}} W_q \left[ \left( p^* - \frac{1}{Re} \frac{\partial u_x^*}{\partial x^*} \right) \psi _{x, k} \right] _{x_{\min }^*}^{x_{\max }^*} J_{y^*} \\&- \sum _{q=1}^{Q_{x^*}} W_q \left[ \frac{1}{Re} \frac{\partial u_x^*}{\partial y^*} \psi _{x, k} \right] _{y_{\min }^*}^{y_{\max }^*} J_{x^*} \end{aligned} \right) \right) ^2. \end{aligned} } \end{aligned}$$
- The momentum equation in the $y^*-$direction loss function
  41 $$\begin{aligned} { \displaystyle \begin{aligned} \mathscr {L}_{M, y}&= \frac{1}{\mathscr {N}_{el}K} \sum _{n=1}^{N_{el}} \sum _{k=1}^{K} \left( \frac{1}{J_{x^*}J_{y^*}}\left( \begin{aligned}&\sum _{q=1}^{Q} W_q \Bigg [ \Big ( u_x^* \frac{\partial u_y^*}{\partial x^*} + u_y^* \frac{\partial u_y^*}{\partial y^*} \Big ) \psi _{y, k} \\&- \frac{1}{J_{y^*}} p^* \frac{\partial \psi _{y, k}}{\partial y^*} + \frac{1}{Re} \left( \frac{1}{J_x^*} \frac{\partial u_y^*}{\partial x^*} \frac{\partial \psi _{y, k}}{\partial x^*} + \frac{1}{J_y^*} \frac{\partial u_y^*}{\partial y^*} \frac{\partial \psi _{y, k}}{\partial y^*} \right) \Bigg ] J_{x^*} J_{y^*} \\&- \sum _{q=1}^{Q_{y^*}} W_q \left[ \frac{1}{Re} \frac{\partial u_y^*}{\partial x^*} \psi _{y, k} \right] _{x_{\min }^*}^{x_{\max }^*} J_{y^*} \\&+ \sum _{q=1}^{Q_{x^*}} W_q \left[ \left( p^* - \frac{1}{Re} \frac{\partial u_y^*}{\partial y^*} \right) \psi _{y, k} \right] _{y_{\min }^*}^{y_{\max }^*} J_{x^*} \end{aligned} \right) \right) ^2. \end{aligned}} \end{aligned}$$

The total residual loss function becomes

42 $$\begin{aligned} \mathscr {L}_{\mathscr {R}} = \mathscr {L}_C + \mathscr {L}_{M, x} + \mathscr {L}_{M, y}. \end{aligned}$$

The NN takes two inputs $(x^*, y^*)$ and gives three outputs $(u_x^*, u_y^*, p^*)$. The subscript *NN* is not used here for convenience.
